# Egy-Score Predicts Severe Hepatic Fibrosis and Cirrhosis in Egyptians With Chronic Liver Diseases: A Pilot Study

**DOI:** 10.5812/hepatmon.10810

**Published:** 2013-06-16

**Authors:** Mohamed A. Alboraie, Mahmoud E. Afifi, Fathy G. Elghamry, Helmy A. Shalaby, Gamal E. Elshennawy, Ahmed A. Abdelaziz, Mohamed U. Shaheen, Amany R. Abo El-Seoud

**Affiliations:** 1 Department of Internal Medicine, Al-Azhar University, Cairo, Egypt; 2 Department of Clinical Pathology, Al-Azhar University, Cairo, Egypt; 3 Department of Community Medicine and Public Health, Zagazig University, Zagazig, Egypt

**Keywords:** Alpha-Macroglobulins, CA-19-9 Antigen, Biological Markers, Fibrosis, Liver Cirrhosis

## Abstract

**Background:**

Non-invasive methods for assessment of hepatic fibrosis are increasingly needed. Recent studies showed that combined elevation of tumor markers CA 19-9 and CA 125 is predictive of severe hepatic fibrosis or cirrhosis with high specificity.

**Objectives:**

We aimed at developing a new panel of surrogate biomarkers for prediction of the stage of hepatic fibrosis by combining tumor markers with other known biomarkers of hepatic fibrosis.

**Patients and Methods:**

A total of 92 patients with different types of chronic liver diseases (chronic hepatitis B, chronic hepatitis C and autoimmune hepatitis), were prospectively enrolled in our cohort. They were subjected to: ALT, AST, GGT, ALP, total bilirubin, INR, total cholesterol, albumin, platelet count, cancer antigen 19-9 (CA 19-9), cancer antigen 125 (CA 125), cancer antigen 15-3 (CA 15-3), haptoglobin, alpha-2-macroglobulin, apolipoprotein A1, abdominal ultrasound, liver biopsy and histological staging of hepatic fibrosis using the METAVIR system.

**Results:**

Combined elevation of CA 19-9 and CA 125 with a summated value > 37 U/mL is predictive of severe hepatic fibrosis or cirrhosis (stage F3-F4 METAVIR) with a probability of 77.6%. Multivariate analysis showed that the most relevant collection of biomarkers for prediction of stage of hepatic fibrosis is: CA 19-9, age, alpha-2- macroglobulin, total bilirubin, platelet count & albumin. We developed a new score, named the “Egy-Score”, using a regression equation composed of this panel of biomarkers. Egy-Score could differentiate no or early fibrosis (stage F0-F2 METAVIR) from severe fibrosis or cirrhosis (stage F3-F4 METAVIR) with 83.7% accuracy.

**Conclusions:**

Non-invasive assessment of hepatic fibrosis could be done using the Egy-Score. Egy-Score could differentiate no or early fibrosis (stage F0-F2 METAVIR) from severe fibrosis or cirrhosis (stage F3 - F4 METAVIR) with 83.7% accuracy.

## 1. Background

Due to the limitations and the invasive nature of liver biopsy, there has been extensive interest in developing non-invasive tests to measure liver fibrosis (1). These are alternatives to liver biopsy that can be used in clinical practice, with benefits in terms of cost, risk, and patient convenience (2). Clinically applicable non-invasive tests include radiological studies, transient elastography (TE), and serum markers. Most noninvasive tests of liver fibrosis were developed with the aim of discriminating between “insignificant”, (F0-F1) by METAVIR and clinically “significant” fibrosis (≥ F2) by METAVIR or for identifying or excluding established cirrhosis in patients with well compensated chronic liver disease. Both these aims are clinically the most relevant (3). Diagnosing or excluding cirrhosis has major implications for patient outcomes and mandates radiological screening every six months for hepatocellular carcinoma and endoscopy to rule out portal hypertension (4). Recent radiological advances allow the bedside assessment of liver stiffness with techniques like: TE (5), acoustic radiation force impulse (ARFI) (6) and magnetic resonance elastography (MRE) (7). TE is currently the most widely used and best validated technique for noninvasive assessment of liver fibrosis worldwide, mainly in viral hepatitis. Its diagnostic performance is better for cirrhosis than for significant fibrosis, with mean area under the receiver operating characteristic curve (AUROC) values of 0.94 and 0.84, respectively (8). TE has also been found as a reliable method for assessment of chronic liver disease of non-viral etiology; such as alcoholic liver disease (9), nonalcoholic fatty liver disease (NAFLD) (10) and cystic fibrosis (11). A variety of direct serum markers of fibrosis, reflecting either the deposition or the removal of extracellular matrix in the liver, have been evaluated for their ability to assess liver fibrosis. They include: glycoproteins such as Alpha-2-Macroglobulin (α2-MG), serum hyaluronate, laminin, and YKL-40; the collagen families such as procollagen III N-peptide and type IV collagen; collagenases and their inhibitors such as matrix metalloproteases and tissue inhibitory metalloprotease-1. Indirect serum markers including simple routine blood tests such as prothrombin index, platelet count, and AST/ALT ratio have also been proposed (12). Recently genomic and proteomic research provides many candidate biomarkers, but independent validation of these biomarkers is lacking, and reproducibility is still a key concern (13). Also various tumor markers in serum including α-fetoprotein, carcinoembryonic antigen, cancer antigen 19-9 (CA 19-9), and cancer antigen 125 (CA 125) have been reported to be elevated nonspecifically in liver disease or nonmalignant conditions (14). The limitations of individual markers to assess liver fibrosis have led to the development of more sophisticated algorithms or indices combining the results of panels of markers that substantially improved diagnostic accuracy for which FibroTest has been the pioneer (12). FibroTest combines five laboratory parameters (α2-macroglobulin, haptoglobin, apolipoprotein A1, γ-glutamyl transpeptidase (GGT), and total bilirubin) in addition to age and sex. Other widely used panels of biomarkers include: The AST-to-Platelet Ratio Index (APRI), FibroIndex (platelet count, AST, GGT), FibroMeter (platelet count, α2 macroglobulin, AST, age, prothrombin index, hyaluroinc acid (HA), blood urea nitrogen), Forns (age, platelet count, GGT, cholesterol levels), Hepascore (age, gender, bilirubin, GGT, HA, α2-macroglobulin) and Fib-4 (platelet count, ALT, AST, platelet count, age) (15). More recent biomarker panels include: Fibro-α score (alpha Feto-Protein, aspartate aminotransferase, aspartate aminotransferase/alanine aminotransferase and platelet count) (16) and FibroSteps (hyaluronic acid, TGF-β1, α2-macroglobulin, MMP-2, apolipoprotein-A1, urea, MMP-1, alpha-fetoprotein, haptoglobin, RBCs, haemoglobin and TIMP-1) (17).

## 2. Objectives

In this study we aimed at exploring the pattern of elevation of tumor markers CA 19-9, CA 125 and CA 15-3 in patients with chronic liver disease of different etiology and to evaluate the usefulness of this elevation as a predictor for the severity of hepatic fibrosis. Moreover we aimed at developing a new panel of biomarkers for assessment of hepatic fibrosis using tumor markers in combination with other known noninvasive markers of hepatic fibrosis.

## 3. Patients and Methods

Our study included ninety two treatment naive patients (48 males and 44 females). They were collected during the period between October 2007 and May 2010 from the outpatient clinics of Al-Hussein hospital, Al-Azhar University. Their mean age was 57.29 ± 13.98 years and they had chronic liver disease of different etiology. Patients were classified in to three initial groups: Group I: twenty six patients with chronic hepatitis B viral infection (CHB), Group II: thirty patients with chronic hepatitis C viral infection (CHC) and Group III: thirty six patients with autoimmune hepatitis (AIH). Demographic data for the studied groups are shown in Table 1. Patients with other causes of liver disease e.g. primary biliary cirrhosis, primary sclerosing cholangitis, Wilson’s disease, and haemochromatosis were excluded. Patients with evidence of hepatic, pancreatic, ovarian or breast tumors were also excluded. The study protocol was approved by the ethical committee of faculty of medicine Al-Azhar University and informed consents were taken from all participating subjects. All patients were subjected to the following laboratory tests: ALT, AST, GGT, ALP, total bilirubin, INR, total cholesterol, albumin and platelet count using standard laboratory techniques. Serum levels of CA 19-9, CA 125, CA 15-3 (using ECLIA/ROCHE Diagnostics; MODULAR ANALYTICS E170), haptoglobin and serum alpha-2-macroglobulin levels using an automatic nephelometer (BNII, Dade Behring; Marburg, Germany) and serum apolipoprotein A1 (Apo-A1) (using ELISA kits, Roche, Switzerland) were also assessed.

**Table 1.** Demographic Data for the Studied Groups

Variables	Total (n = 92)	Group I, HBV (n = 26)	Group II, HCV (n = 30)	Group III, AIH (n = 36)	P value
**Age, y, range, mean ± SD**	18–82, 57.29 ± 13.98	23–82, 61.04 ± 16.63	28–72, 55.2 ± 12.42	18–79, 56.33 ± 13.2	0.2607
**Males No. (%)**	48 (52.2)	21 (80.8)	18 (60)	9 (25)	
**Females No. (%)**	44 (47.8)	5 (19.2)	12 (40)	27 (75)	< 0.001
**Fibrosis stage, No. (%)**					0.0635
F0	14 (15.2)	2 (7.7)	6 (20)	6 (16.7)	
F1	20 (21.7)	4 (15.4)	5 (16.7)	11 (30.6)	
F2	15 (16.3)	3 (11.5)	2 (6.7)	10 (27.7)	
F3	10 (10.9)	4 (15.4)	4 (13.3)	2 (5.6)	
F4	33 (35.9)	13 (50)	13 (43.3)	7 (19.4)	

### 3.1. Abdominal and Pelvic Ultrasound

Abdominal and pelvic ultrasounds were done to assess the severity of liver disease and to rule out presence of hepatic, pancreatic or ovarian lesions.

### 3.2. Liver Biopsy

Ultrasound guided percutaneous liver biopsy specimens were taken from the patients and examined by two different pathologists (M. A. and H. Kh.), experienced in liver histology, who were unaware of the laboratory results or clinical diagnosis. Only specimens with inter-observer agreement of stage of hepatic fibrosis were included in the study. METAVIR scoring system was used for staging hepatic fibrosis. Every biopsy specimen was staged on a scale of F0 to F4 (F0 = no fibrosis, F1 = portal fibrosis without septa, F2 = few septa, F3 = numerous septa without cirrhosis, and F4 = cirrhosis) (18, 19).

### 3.3. Statistical Analysis

Sample size was calculated using the Epi-Info statistical software (version 6.04, WHO, 2001). Sample size was determined based on the expected prevalence of liver fibrosis in the population in outpatient clinic (35%) and the worst accepted was 25%. At 95% confidence level, the sample was 87 and an additional 10% were selected to compensate for refusals or patients not fulfilling the criteria (8 patients). Three patients were excluded from the analysis due to inter observer variability in liver biopsy specimen’s histopathological examination. The three patient groups were sub classified into 5 fibrosis strata: F0, F1, F2, F3 and F4 and four main comparison groups (F0-F1, F0-F2, F2-F4 and F3-F4) according to the stage of fibrosis in the liver biopsy by MEAVIR. After collecting date, we reviewed the data by examining minimum and maximum values in each variable to detect any abnormal or outlier values. Then we summarized the data using descriptive statistics. We examined the effect of each single variable on outcome, as no/mild (F0-F1), early (F0-F2) significant (F2-F4) or severe fibrosis/cirrhosis (F3-F4). We examined different cutoff points for each variable to get the strongest one. Then we checked correlations, sensitivity, specificity and predictive values between several variables and outcome. Previous steps gave us a good idea about the importance of variables. We then examined confounding variables by using logistic regression to develop a model for predicting stage of hepatic fibrosis. The tests used for statistical analysis included; X mean and SD standard deviation, to measure the central tendency of data and the distribution of data around their mean value; student’s t-test for testing statistical significant differences between mean values of two samples; X2 test (Chi square test), to test for statistically significant relationships between different variables or grades in qualitative data; ANOVA or F test, to test for significant differences between more than two sample mean values; Pearson correlation coefficient test, (r) to test for linear relationships between two numeric variables; Mann Whitney test: non parametric test for comparing two groups of data not normally distributed or for small sample size; multiple regressions, to analyze a single dependent variable, Y ,that is of interest to predict one or more independent variable, X, that explain the variations that occur in Y; and Fisher exact test, for comparing two independent proportions when the expected observation in any cell of the table is below 5. Results were considered as significant, if P < 0.05 and highly significant if P < 0.01.

## 4. Results

Our results indicated that there was a highly significant positive correlation between stage of fibrosis and age, alpha-2-macroglobulin, bilirubin, AST, alkaline phosphatase (ALP), GGT, CA 19-9, CA 125, CA 15-3 and INR. On the other hand, fibrosis stage was negatively correlated with haptoglobin, cholesterol, albumin, Apo A1 and platelets. There was no significant linear correlation between fibrosis stage and ALT (Table 2). There were highly significant statistical differences between cases with different fibrosis stages regarding age, alpha-2-macroglobulin, bilirubin, cholesterol, AST, ALP, GGT, albumin, Apo A1, INR and platelet count. Cases with fibrosis stage F4, for example, were older; they recorded higher readings of alpha-2-macroglobulin, total bilirubin and AST. They also had lower values of haptoglobin and cholesterol. There were highly significant statistical differences between cases with different fibrosis stages regarding mean values of CA 19-9 and CA 125. There was no significant difference between groups in mean value of CA 15-3 (Table 3).

**Table 2.** Correlation Between Fibrosis Stages and the Studied Variables

Correlation	r	P value
**Fibrosis stage with age**	0.44	< 0.01
**Fibrosis stage with ** **haptoglobin**	-0.28	< 0.01
**Fibrosis stage with alpha 2 ** **macroglobulin**	0.41	< 0.01
**Fibrosis stage with bilirubin**	0.48	< 0.01
**Fibrosis stage with cholesterol**	-0.33	< 0.01
**Fibrosis stage with ALT**	0.06	> 0.05
**Fibrosis stage with AST**	0.35	< 0.01
**Fibrosis stage with ALP**	0.28	< 0.01
**Fibrosis stage with GGT**	0.37	< 0.01
**Fibrosis stage with albumin**	-0.46	< 0.01
**Fibrosis stage with Apo A1**	-0.34	< 0.01
**Fibrosis stage with CA 125**	0.32	< 0.01
**Fibrosis stage with CA 19-9**	0.38	< 0.01
**Fibrosis stage with CA 15-3**	0.25	< 0.05
**Fibrosis stage with INR**	0.42	< 0.01
**Fibrosis stage with Platelet count**	-0.59	< 0.01

**Table 3.** Mean Values ± SD of Different Variables with Different Fibrosis Stages (METAVIR) ^a^

Variables	Stage F0 (n = 14)	Stage F1 (n = 20)	Stage F2 (n = 15)	Stage F3 (n = 10)	Stage F4 (n = 33)	P value
**Age, y, Mean ± SD**	43.21 ± 14.49	54.3 ± 14.54	60.47 ± 14.47	58.9 ± 8.83	63.15 ± 9.92	< 0.001
**Haptoglobin, g/l, Mean ± SD**	1.22 ± 0.46	1.42 ± 0.57	1.5 ± 0.73	1.33 ± 1.26	0.78 ± 0.77	0.009
**α2-MG, g/l, Mean ± SD**	2.07 ± 0.66	2.31 ± 0.91	2.39 ± 0.71	2.77 ± 0.97	3.04 ± 0.88	0.002
**Total bilirubin, umol/l, Mean ± SD**	7.8 ± 4.01	9.02 ± 4.78	8.85 ± 4.81	10.03 ± 5.19	19.39 ± 11.97	< 0.001
**Cholesterol, mmol/l, Mean ± SD**	5.22 ± 1.38	5.5 ± 1.13	5.54 ± 1.37	4.98 ± 0.58	4.37 ± 1.18	0.004
**ALT, U/L, Mean ± SD**	56 ± 37	62 ± 43	54 ± 0.34	77 ± 59	62 ± 41	0.710
**AST, U/L, Mean ± SD**	58 ± 26	59 ± 25	53 ± 0.16	67 ± 31	86 ± 41	0.003
**ALP, U/L, Mean ± SD**	121 ± 57	135 ± 36	147 ± 56	153 ± 55	237 ± 246	0.006
**GGT, U/L, Mean ± SD**	78 ± 99	99 ± 118	95 ± 81	104 ± 67	293 ± 324	0.001
**Albumin, g/l, Mean ± SD**	45.91 ± 3.11	45.83 ± 3.32	44.63 ± 1.99	44.62 ± 3.08	40.35 ± 5.95	< 0.001
**Apo A1, g/l, Mean ± SD**	1.72 ± 0.38	1.69 ± 0.39	1.89 ± 0.55	1.53 ± 0.30	1.39 ± 0.33	0.009
**INR, Mean ± SD**	0.93 ± 0.02	0.94 ± 0.04	0.94 ± 0.03	0.94 ± 0.03	1.05 ± 0.16	< 0.001
**Platelet, k/ul, Mean ± SD**	287.36 ± 68.56	284.15 ± 18.07	257.8 ± 56.31	195.5 ± 74.15	155.03 ± 86.5	< 0.001
**CA 19-9, U/ml, Mean ± SD**	6.81 ± 4.49	10.6 ± 7.95	30.89 ± 43.6	40.08 ± 52.34	37.37 ± 30.03	0.002
Median	5.95	8.2	23.1	24.5	28.8	< 0.001
> 27 (%)	0	1 (5)	6 (40)	5 (50)	20 (61)	< 0.001
**CA 125, U/ml, Mean ± SD**	14.36 ± 8.68	16.05 ± 10.17	17.45 ± 9.77	19.36 ± 12.56	128.57 ± 126.7	<0.001
Median	12.75	11.1	12.7	17.95	32.8	< 0.001
> 35 (%)	1 (7.1)	1 (5)	2 (13.3)	1 (10)	16 (48.5)	< 0.001
**CA 15-3, U/ml, Mean ± SD**	16.75 ± 6.05	19.42 ± 12.39	20.87 ± 6.39	23.24 ± 9.87	25.12 ± 15.43	0.204
Median	15.55	17.6	21.4	20.4	21.1	0.047
> 25 (%)	2 (14.3)	3 (15)	2 (13.3)	3 (30)	12 (36.4)	0.237

^a^ Normal ranges: Haptoglobin (0.30 - 2.00 g/l), α2-MG (1.30 - 3.00 g/l), total bilirubin (< 21.0 umol/l), cholesterol (< 5.2 mmol/l), ALT 17 - 83 U/L (for males), ALT 17 - 58 U/ L (for females), AST 17-83 U/L (for males), AST 17-58 U/L (for females), ALP 67 - 215 U/ L (for males), ALP 58 - 174 U/ L (for females), GGT 17 - 119 U/ L (for males), GGT 10 - 70 U/ L (for females), Albumin 35.0 - 52.0 g/l, Apo A-1 1.10-1.60 g/l, INR < 1.25, Platelet count 150 - 450 k/ul, CA 19-9 < 27 U/ml, CA 125 < 35 U/ml, CA 15-3 < 25 U/ml.

### 4.1. Correlation Between Tumor Markers and Other Variables in the Studied Groups

There was a significant positive correlation between CA 19-9 and age, total bilirubin, ALT, AST, GGT, CA 15-3, CA 125, and fibrosis stage. There was a significant negative correlation between CA 19-9 and total cholesterol, albumin, and platelet count. There was a significant positive correlation between CA 125 and total bilirubin, ALP, GGT, CA 15-3, CA 19-9, INR, and fibrosis stage. There was a significant negative correlation between CA 125 and total cholesterol, albumin, Apo A1 and platelet count.

### 4.2. Sensitivity, Specificity and Predictive Values of Tumor Markers CA 19-9 and CA 125 in Detection of Hepatic Fibrosis 

**Figure 1.**
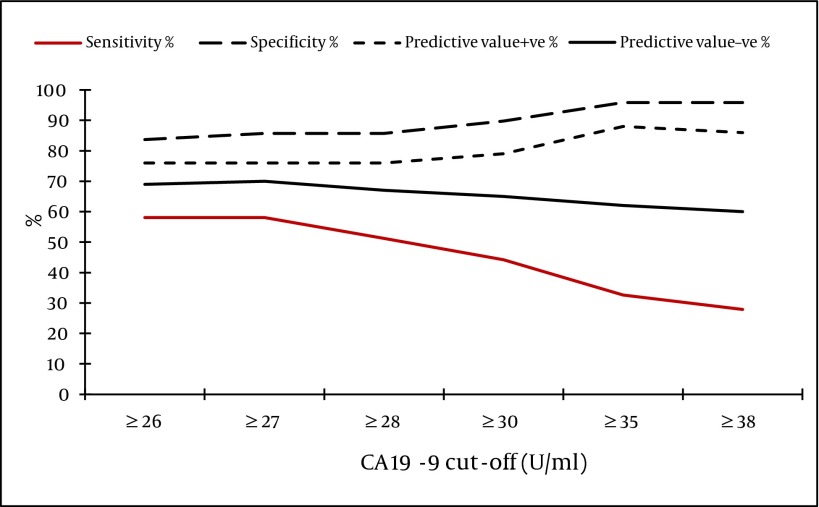
Sensitivity, Specificity and Predictive Values of CA 19-9 in Detection of Severe Hepatic Fibrosis: (Fibrosis Stages F0-F2 Versus Fibrosis Stages F3-F4 by METAVIR)

**Figure 2.**
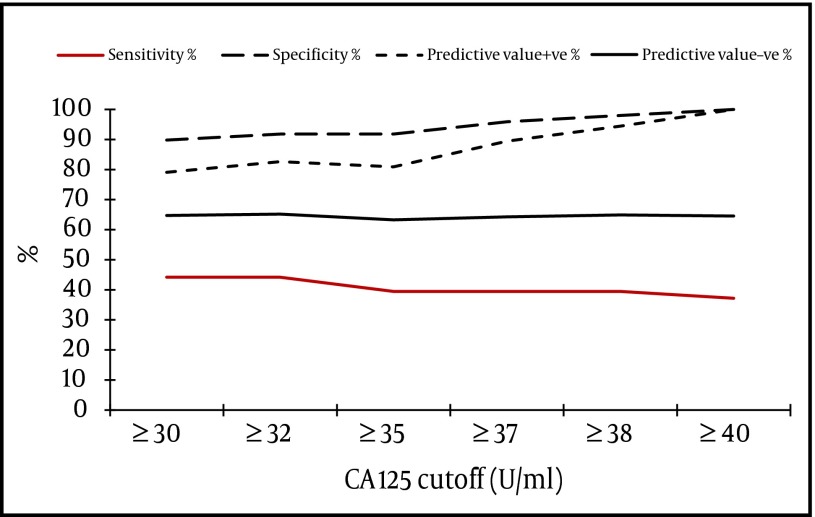
Sensitivity, Specificity and Predictive Values of CA 125 in Detection of Severe Hepatic Fibrosis: (Fibrosis Stages F0-F2 Versus Fibrosis Stages F3-F4 by METAVIR)

We analyzed the levels of tumor markers CA 19-9 and CA 125 in relation to the stage of hepatic fibrosis by METAVIR, aiming at obtain the best cut-off level that gives the best sensitivity and specificity for prediction of the stage of hepatic fibrosis and we found that:

- The best cut-off level of CA 19-9 for detection of severe fibrosis/cirrhosis (stage F3-F4) was ≥ 35 U/ml; 95.9% of cases with CA 19-9 levels below 35 U/ml were in stage F0-F2 (Figure 1).

- The best cut-off level of CA 125 for detection of severe fibrosis/cirrhosis (stage F3-F4) was ≥ 40 U/ml; 100% of cases with CA 125 levels below 40 U/ml were in stage F0-F2 (Figure 2). When both tumor markers were combined together we found that the best cutoff level of summated values of CA 125 and CA 19-9 for detection of fibrosis stage F0-F2 is < 37 U/ml (Sensitivity = 77.6%, Specificity = 69.8%, PVP = 74.5% and PVN = 73.2%) (Table 4), i.e. for any patient with suspected hepatic fibrosis; we summate his CA 125 and CA 19-9 serum levels, if the product is < 37 U/ml, the patient will be in stages < F3 with a probability of 77.6%. If another patient has a CA 125 + CA 19-9 value > 37 U/ml, he is at F3-F4 stage of fibrosis with a probability of 69.8%.

**Table 4.** Sensitivity, Specificity and Predictive Values of CA 125 + CA 19-9 in Detection of Significant Fibrosis

CA 125 + CA 19-9	Fibrosis stage F0-F2	Fibrosis stage F3-F4	Total
**< 37, U/ml**	38	13	51
**≥ 37, U/ml**	11	30	41
**Total **	49	43	92

### 4.3. Development of a Panel of Biomarkers for Prediction of Stage of Hepatic Fibrosis 

Multiple regression analysis for prediction of the stage of hepatic fibrosis indicated that CA 19-9, age, alpha-2-macroglobulin, total bilirubin, platelet count and albumin were the most relevant biomarkers related to the stage of hepatic fibrosis (Table 5). Using logistic regression analysis; a new panel for the prediction of severe hepatic fibrosis was created using these relevant biomarkers. The regression equation (Egy-Score) for the detection of the stage of fibrosis was:

Fibrosis stage (Egy-Score) = 3.52 + 0.0063 x CA 19-9 (U/ml) + 0.0203 x age (year) + 0.4485 x alpha-2-macroglobulin (g/l)+ 0.0303 x bilirubin (umol/l) – 0.0048 x platelet (K/uL) – 0.0462 x albumin (g/l).

Prediction of the stage of hepatic fibrosis by the Egy-Score has 79.3% accuracy considering fibrosis stages F0-F1 as a group and stages F2-F4 as another group. Sensitivity of the Egy-Score in detection of stages F0-F1 is 79.4% and its specificity is 79.3%. Percentage of cases with stage F2- F4 who were diagnosed as stages F0-F1 were 12/58 = 20.6% (false positive). Accuracy of Egy-Score = 27+46 / 92 =79.3%. Prediction of the stage of hepatic fibrosis by Egy-Score is 83.7% accurate considering fibrosis stages F0-F2 as a group and stages F3-F4 as another group. Sensitivity of the Egy-Score in detection of stages F0-F2 is 95.9% and specificity is 69.8%. Percentage of cases with stages F3-F4 who were diagnosed as stages F0- F2 was 13/43 = 30.2% (false negative).

Accuracy of Egy-Score = 47+30 / 92 = 83.7%. It is better to include stages F0- F2 in one group and stages F3-F4 in another group as it gives a more accurate prediction. The score of the equation (Egy-Score) ranges from 1-5 and it correlates to F0-F4 degree of fibrosis by METAVIR; the cut off values for significant fibrosis (≥ F2 METAVIR) is ≥ 3 and for severe fibrosis/cirrhosis (≥ F3 METAVIR) is ≥ 4.

**Table 5.** Multiple Regression Analysis for Prediction of Different Stages of Hepatic Fibrosis by Using the Most Relevant Variables

Variables	B coefficient	95% CI		SE	F	P value
		Lower limit	Upper limit			
**CA 19-9**	0.0063	-0.0006	0.0133	0.0035	3.24	0.01
**Age**	0.0203	0.0038	0.0369	0.0083	5.97	0.001
**Alpha-2 macroglobulin**	0.4485	0.1971	0.6999	0.1264	12.58	< 0.001
**Bilirubin **	0.0303	0.0022	0.0583	0.0140	4.6278	0.01
**platelet**	-0.0048	-0.0074	-0.0021	0.0013	13.06	< 0.001
**Albumin **	-0.0462	-0.1006	0.0082	0.0274	2.842	0.05
**Y intercept**	3.52					

## 5. Discussion

Although, liver biopsy is still considered as the gold standard for identifying liver histological stages, an assessment of the disease development based on non-invasive clinical findings is also emerging and this may replace the need of biopsy in the near future. Even though from it’s beginning, TE was proved to be best with high AUROCs (> 0.90) in all studies, no single noninvasive marker has been able to differentiate all fibrosis stages from end stage cirrhosis. Non-invasive biomarkers like: FibroTest, Forn's Index, Fibrometer and HepaScore have high five-year predictive values but with low AUROCs (0.60~0.85) and are not comparable to liver biopsy (AUROC = 0.97). There is still a need of a marker which accurately determines the stage based on simplest routine laboratory test (20). In this study we aimed at exploring the role of elevated serum tumor markers as predictors of the stage of hepatic fibrosis and to combine them with known biomarkers of liver fibrosis to form a new score that can predict different stages of liver fibrosis. We developed a new score (Egy-Score) that can non-invasively predict significant hepatic fibrosis (≥ F2) with 79.3% accuracy and advanced hepatic fibrosis/ cirrhosis (≥ F3) with 83.7% accuracy.

The score is composed of six biomarkers (CA 19-9, age, alpha-2- macroglobulin, total bilirubin, platelet count & albumin), which are previously known to have significant diagnostic values of staging hepatic fibrosis and progression of liver disease in general (14, 21-25). Although there are several models described to predict severe liver fibrosis on the basis of laboratory parameters; either free to use like APRI (25) and Forns (23) or protected by patents like: the FibroTest (26), Fibrometers (27), FibroSpect (28), Enhanced Liver Fibrosis Test (ELF) (29, 30) and HepaScore (31); none of them have described the use of tumor markers for prediction of liver fibrosis, even recently developed models including: Fibro-α Score (16) and FibroSteps (17). Most of these models were validated in chronic hepatitis C; few are validated for other causes of chronic liver disease. When compared and validated externally in patients with hepatitis C, the different patented scores have similar performances for the diagnosis of significant fibrosis (32). In a large study (n = 1,307) (33), comparing prospectively several patented and non-patented scores (FibroTest, Fibrometre, Hepacore and APRI), the AUROCs ranged from 0.72 to 0.78 for significant fibrosis and from 0.77 to 0.86 for cirrhosis. Although non-patented scores, such as the Forns index, FIB-4, and APRI may have slightly lower performances, they are cost-free, easy to calculate, and available almost everywhere (32). Fibro-α score predicts significant liver fibrosis (F2–F4) with 70% sensitivity and 60% specificity, advanced liver fibrosis (F3–F4) with 88% sensitivity and 60% specificity and liver cirrhosis (F4) with 90% sensitivity and 57% specificity (16). In a recent study, FibroSteps (17) could differentiate different stages of hepatic fibrosis with accuracies of 94.9% and 89.8% in the training and validation sets, respectively. Although FibroSteps results are encouraging; it has the disadvantage of combining twelve parameters, nine of which are not routinely requested and this may be coasty to many users. On the other hand, extensive published data have demonstrated the ability of TE to predict the degree of hepatic fibrosis and to have excellent (94%) diagnostic accuracy for cirrhosis (11) in addition to its value for the prediction of clinically significant portal hypertension and diagnosis of varices (34). Therefor; Egy-Score performance in prediction of significant fibrosis (≥ F2) and advanced fibrosis (≥ F3) should be tested against the widely accepted non-invasive methods for assessing hepatic fibrosis and cirrhosis including biomarker panels & TE in progressively enrolled cohorts. Limitations of our study includes: elevation of the tumor markers have been associated with the presence of ascites (35) or cholestasis (36, 37) in liver disease patients and this may give false positive results for our scores. The study was conducted on a small number of patients and it needs validation with a larger number of patients in a prospectively enrolled cohort. Our scores depend mainly on simple routinely used laboratory parameters (total bilirubin, albumin, platelet count) in addition to age and 2 non routine tests (CA 19-9 and Alpha-2-Macroglobulin). Although this panel needs to be done in validated laboratories, the cost of our score is much cheaper than other well-known and patented tests such as FibroTest and FibroMetrers.

Tumor markers are frequently elevated in patients with chronic liver diseases especially CA 19-9, CA 125 and less frequently for CA 15-3. CA 19-9 and CA 125 can be used as markers for hepatic fibrosis either alone or in combination. Both significant and advanced hepatic fibrosis could be predicted by a novel panel of serum biomarkers (Egy-Score) composed of CA 19-9, age, alpha-2- macroglobulin, total bilirubin, albumin and platelet count (in a regression equation) with good sensitivity and specificity. Egy-Score can be applied easily in clinical practice to exclude severe hepatic fibrosis/cirrhosis in patients with contraindication for liver biopsy or those who are reluctant to do it. Egy-score would need further validation to be regarded as an alternative to liver biopsy. One should be careful when interpreting elevated levels of tumor markers CA 19-9 and CA 125 in patients with chronic liver disease as this could be a benign elevation related to hepatic fibrosis and not necessarily due to underlying malignancies.
